# The impact of work-related foot pain on work and personal life: an interview study

**DOI:** 10.1186/s12891-026-10624-3

**Published:** 2026-10-14

**Authors:** Ann-Sophie Hörnell, Anncristine Fjellman-Wiklund, Åsa Svedmark, Eva Tengman

**Affiliations:** Dept. of Community Medicine and Rehabilitation, Umeå University, Umeå, SE-90187 Sweden

**Keywords:** Workplace, Workload, Musculoskeletal pain, Foot diseases, Qualitative research

## Abstract

**Background:**

The occurrence of foot pain is high, especially in those with physically demanding occupations. Work-related foot pain has been shown to impact work performance, family life, and leisure activities. While prevalence across various sectors is documented, research exploring experiences and total life impact of work-related foot pain remains limited.

**Aim:**

The aim of this study was to explore experiences of living with foot pain among individuals employed in highly demanding physical occupations, and occupations characterised by prolonged standing or walking.

**Methods:**

A qualitative, semi-structured interview-based approach was chosen. Twelve individual interviews were conducted with nine women and three men, and analysed using Qualitative Content Analysis.

**Results:**

One main theme, *Walking a fine line - Balancing challenges in multiple aspects of life due to work-related foot pain*, and three sub-themes were identified, describing how external work-environment factors and internal experiences of autonomy impact an individual’s overall life situation, including impact on family life and the mental toll of living and working with foot pain. The sub-theme *Struggling with increased pain due to work conditions and environment* highlights how factors such as certain types of floors, physical work demands, time spent on the feet, walking, heavy lifting, and inadequate footwear directly exacerbate pain. The sub-theme *Feeling like just another cog in the machine due to limited control over workplace organisation* reflects difficulties for employees in influencing work tasks, planning daily work tasks and responsibilities, a lack of recovery during the workday, and communication within the workplace. The sub-theme *Experiencing inequality and low quality of life outside of work* describes the consequences for individuals’ personal lives, including restricted social participation, strained family relations, negative emotions, and sleep disturbances. Overall, the individuals expressed a sense of resignation, feeling that they had to “grit their teeth” and continue working despite the pain.

**Conclusion:**

The central findings of this study are negative experiences and struggles, during both work and leisure time. Understanding the experiences of individuals with work-related foot pain can help employers and healthcare providers to identify work-related risks and consider preventative measures, although further research on this topic is needed.

**Clinical trial number:**

Not applicable.

**Supplementary Information:**

The online version contains supplementary material available at https://doi.org/10.1186/s12891-026-10624-3.

## Background

The occurrence of foot pain is relatively high, with population-based studies showing that 17–42% suffers from discomfort and pain in the foot [1–3]. Factors associated with a higher risk of foot disorders and pain include a high body mass index, older age, women sex, previous foot injury or pain, footwear, and with prolonged standing or walking [2, 4–6]. Foot pain is often accompanied by knee, hip, and lower-back pain. Women are more likely to report discomfort in the feet than men, and individuals with lower educational background reports more discomfort and pain than those with higher educational background [5, 7, 8]. The prevalence of foot pain is high in professions that are characterised by high physical demands on the feet, i.e. long periods of walking or prolonged standing, with some occupations reporting prevalences of foot pain of up to 85.5% [9–11]. Individuals suffering from pain in any part of the foot and ankle often experience limitations in participating in their normal activities, such as walking, running, prolonged standing, walking downstairs, and physical activity in general [12–16]. The loss of mobility also affects individuals’ sense of independence, as they often require help in daily-life activities such as housework [12–14]. Even recreational activities such as going on holiday or interacting with family can be impacted [12, 14]. The pain may also lead to difficulty participating in social activities, which often leads to a feeling of social isolation [12, 15] or a loss of identity [16]. Foot pain and the subsequent loss of activity are often linked to negative emotions, low mood, fear of physical activity, depression, anxiety, frustration and trouble sleeping [12–15]. Chronic foot pain can lead to a host of wider problems, such as disability, depression, and stress, along with inability to work and other consequences in relation to work [17].

Work-related musculoskeletal disorders (WMSDs) are defined as pain experienced in direct relation to work, and can have a negative impact on work performance. They can be caused by work requiring a fixed position, repetitive movement, concentrated force on one part of the body, insufficient recovery, or a combination of these factors. WMSDs develop gradually, and are often caused by overuse. They may also affect people’s ability to participate in work [18, 19]. Studies show that many people with foot pain and an occupation that involves prolonged standing, walking, or heavy lifting feel that they cannot meet their work requirements [12–16]. This can lead to sick leave and lower income [15]. Besides work, individuals with foot pain feel that they cannot participate in normal daily activities. At present, research into how foot pain in general is experienced in relation to work, as well as in the ways in which work tasks are impacted, is limited.

The prevalence of foot pain in various professional groups is well documented, and studies of work-related foot pain report correlations between prolonged standing on hard surfaces during work and plantar heel pain [7, 8, 20-21–22]. Studies across various occupational fields show that foot and ankle pain are common in individuals whose work involves prolonged standing, walking, and heavy lifting. For example, 49.5% of hairdressers report experiencing foot pain [8], and 43.3% of farmers report pain in the feet and other parts of the lower body, often associated with a heavy workload and heavy lifting [7]. Healthcare professionals such as nurses also report a high prevalence (51.8%) of foot and ankle pain [21]. These prevalences of foot and ankle pain indicate a health problem across a wide variety of professional fields, educational backgrounds, and socio-economic standings. Although some of the studies mentioned above focus on how different aspects of daily life are affected by foot pain, the topic remains relatively under-researched, particularly with respect to the day-to-day work environment.

Further, few studies have examined which aspects of specific occupations may increase or alleviate individuals’ pain, and how people experience their work environments, participation in work, and activity levels in relation to their foot pain. There may be additional aspects that individuals consider important in relation to foot pain and its impact on work. A deeper understanding of how individuals perceive their foot pain, and how it affects their daily and occupational lives, could help employers and healthcare professionals to better recognise all aspects of the limitations that people experience. This knowledge may contribute to preventing recurrence, improving education, and supporting person-centred, tailored interventions in both healthcare and work-life contexts.

### Aim

The aim of this study was to explore experiences of living with foot pain among individuals employed in highly physically demanding occupations and occupations characterised by prolonged standing or walking.

## Methods

### Study design, recruitment, and participants

This qualitative, inductive study is part of a larger ongoing project that aims to identify the key determinants of work-related foot and ankle disorders associated with prolonged standing and walking. This study utilised individual interviews, which were analysed with Qualitative Content Analysis (QCA) [23]. According to Graneheim and Lundman, a qualitative approach with QCA can showcase lived experiences and phenomena – in this case relating to foot pain – rather than focusing on quantitative data. QCA emphasises the identifying of similarities and differences within and between codes and categories [23]. The study was reported in accordance with the COnsolidated criteria for REporting Qualitative research (COREQ) checklist.

Purposive sampling [24] was used to recruit women and men aged 18–65 years with foot pain that was caused or exacerbated by work. People with various occupations were recruited; all employments had been ongoing for at least one year, and the work was characterised by intense and high physical work demands. Individuals with underlying health conditions such as diabetes or a neurological pathology that posed an increased risk of foot pain were excluded, on the basis that the experienced pain might not be work-related. Individuals who met the inclusion criteria and had recently been in contact with a physiotherapist at a healthcare facility in northern Sweden were asked to participate by the first or last author. Additionally, a few participants were recruited by verbal advertisement by the first author or other participants.

Twelve individuals – nine women and three men – were invited to participate, and all agreed. The participants had not previously been treated by any of the authors, nor were there any personal connections. The recruitment was concluded after twelve interviews as we considered the material as sufficiently rich for further analysis.

This study followed the principles outlined in the Helsinki Declaration [25]. The participants received oral and written information about the study, and provided informed consent in connection with the interview. After the participants had given their consent, they answered a questionnaire regarding background characteristics and foot problems. Questions were asked regarding age, gender, occupation, years of employment at their current workplace, and perceived pain localisation, causation, duration, and level, along with any other health conditions affecting foot pain. Pain intensity was assessed using a 0–10 visual analogue scale (VAS). The VAS for pain intensity has demonstrated good psychometric properties [26]. Self-reported Foot and Ankle Score (SEFAS) was used for foot-specific questions. SEFAS includes 12 questions, each with five answer options; these are combined to calculate a total score ranging from 0 points to 48. Lower scores signify more serious physical limitations, while higher scores indicate less physical limitation. The Swedish version of SEFAS has been found to have acceptable validity and reliability [27].

### Data collection

Data were collected using semi-structured face-to-face or digital interviews [28]. The interviews were conducted at a location of the participants’ choice. All interviews were conducted with the help of an interview guide (see Supplementary), with follow-up questions developed by taking inspiration from the World Health Organizations International Classification of Function, Disability and Health (ICF) [29]: Function, Activity, Participation, Environment, Personal Factors, and Non-Categorised Questions, relating to e.g. whether the participant had sought treatment or knowledge from colleagues with similar problems.

Each interview started with the same open-ended question: “Can you describe your foot pain and how it relates to your work?” During the development of the interview guide, a total of three pilot interviews were conducted, which guided the research group to refine the interview questions. Following the third interview, the interview guide was deemed to be optimal for the aim of the study, and was not changed thereafter. The pilot interviews were not included in the analysis. Four interviews were conducted by the first author (AH), a physiotherapist with clinical experience in primary healthcare and a background in occupational therapy; two interviews were conducted by ET, a physiotherapist with clinical experience in primary healthcare and physiotherapy education; and six interviews were conducted by physiotherapy students in the final year of their education. Participants were given the choice of being interviewed either digitally or face-to-face. Eleven interviews were conducted face-to-face, and one interview was conducted digitally, via Zoom. The interviews lasted 20–45 min, and were audio-recorded. The interview recordings were transcribed verbatim by each person who had conducted the interview. The transcribed material was then compared to the audio recording, and corrected when needed.

### Data analysis

The analysis began with repeated readings of the transcripts by the author (AH) to gain an overall understanding of the content. Short notes were taken, by hand, about the content in the transcripts to help deduct additional meaning of what was said in the interviews. Through the analysis meaning units were identified, condensed, and assigned shorter codes describing the content. The codes were grouped into categories and sub-themes based on content that shared communalities. In the final stage the underlying meaning of the sub-themes was interpreted to one overarching main theme that expressed the latent content of the interviews. Interview transcripts were managed using Microsoft Word, and Microsoft Excel was used to organize, sort, and compare codes and categories throughout the analytical process. Two interviews were first divided into meaning units and coded separately and then discussed and compared by AH and ET. The remaining interviews were coded by AH and clustered into categories. Emerging categories, sub-themes and a main theme were compared, discussed and negotiated between all authors until consensus was reached, to ensure a reliable analysis of the data [30]. To further increase trustworthiness of the study, triangulation between researchers was used [31]. All authors contributed with different experiences and perspectives through their backgrounds in various fields; physiotherapy, rehabilitation, occupational and environmental medicine, behavioral change, long-term pain and stress and mental health.

## Results

Twelve participants, aged 24–58, were interviewed (see Table 1). Two participants worked in a factory, three in healthcare, one in a care facility for the elderly, one in childcare, two in restaurants, one as a mechanic in a workshop, and two as janitorial and cleaning professionals. The participants averaged seven hours on their feet during their work hours. Work experience in their fields ranged from one to 30 years. Some experienced pain in both feet, others predominantly in one foot. The individual pain levels experienced by the participants in the week preceding their interviews ranged from 2.4 to 9.9 on a 0–10 visual analogue scale, and they rated SEFAS from 17 to 45. Foot pain was mostly experienced in the heels or ankles; some participants reported pain in the footpads or lateral side of the foot. The participants experienced pain durations ranging from one to 25 years.

**Table 1:** Patient characteristics

Gender	Age	Profession	Years in profession	Hours spend on the feet	Total daily steps	Years of pain	Current pain^a^	Highest pain in last week^b^	SEFAS score	
W	57	Healthcare	30	6	7000–9000	1	4.7	3.9	25	
M	45	Factory	19	8	12,000–15,000	25	1.9	2.4	24	
W	47	Factory	1	2–6	8000	15	1.8	5.5	22	
W	50	Cleaner	32	8	18,000	2.5	9.6	9.9	17	
M	26	Restaurant	10	8–13	8500	4	1.7	6	34	
W	58	Healthcare	18	4	8000	1	1.6	2.9	41	
W	57	Education	20	7	-	10	4.6	5.9	45	
M	33	Security	13	4	6500	10	4	4.7	40	
W	24	Education	2.5	6	12,000–16,000	10	6.9	7.50	35	
W	25	Restaurant	1.5	8–13	8500	2	0	9.90	27	
W	60	Healthcare	30	10	5500	1	7.9	8.2	26	
W	50	Cleaner	30	7	10,000	1	2.4	7.8	31	

W=Women, M = Men ^a−b^ Pain intensity was assessed using a 0–10 visual analogue (0–10) no pain to extremely high pain,

SEFAS - Self-reported Foot and Ankle Score

A main theme – *Walking a fine line - Balancing challenges in multiple aspects of life due to work-related foot pain* – was identified, as were three sub-themes: *Struggling with increased pain due to work conditions and environment*, *Feeling like just another cog in the machine due to limited control over workplace organisation*, and *Experiencing an uphill battle due to inequality and low quality of life outside of work.* Each sub-theme was connected to two categories (see Table 2).

The results are presented in relation to the main theme, as a short overview, and elaborated in more detail one sub-theme at a time, with the categories summarised in the text. Quotations are presented in some cases with “I” for interviewer and “P” for participant, when both the question and answer are necessary for context. Ellipses are used when a quote was edited for brevity, and square brackets are placed around words that were changed from the original to protect the participants’ anonymity.

**Table 2:** The theme and subthemes that emerged during the analysis

Categories	Sub-theme	Theme
Factors in the work environment that negatively impact foot pain	Struggling with increased pain due to work conditions and environment	Walking a fine line - Balancing challenges in multiple aspects of life due to work-related foot pain
Physical demands and conditions at work that negatively impact foot pain		
Limited ability to influence work	Feeling like just another cog in the machine due to limited control over workplace organisation	
Employer and colleagues as both help and hindrance		
Negative implications of foot pain for life outside of work	Experiencing an uphill battle due to inequality and low quality of life outside of work	
Available help for foot pain		

### Walking a fine line- balancing challenges in multiple aspects of life due to work related foot pain

The main theme of *Walking a fine line- Balancing challenges in multiple aspects of life due to work related foot pain* captures the participants’ experience of trying to manage the underlying frustration or resignation to living with foot pain, while still expressing the desire to “make things work” despite this, throughout multiple aspects of work and life. This includes external factors – aspects of work or life that the participants could not actively influence, such as work environment – as well as internal factors – where the participants experienced autonomy and the ability to change aspects of their work and perceived hindrances to achieving change. Both external and internal factors were perceived as being connected to the experienced pain in the feet, including experience and expectations of medical treatment, impacts on family life, and the mental toll of living and working with foot pain.

#### Struggling with increased pain due to work conditions and environment

The participants often identified aspects of their work environments that they believed caused or increased their foot pain. Hard floors made of concrete, stone, or tile often place a considerable stress on the feet, and were felt to have a negative effect – especially for participants who primarily worked standing on such types of floors for most of their workdays. Others found uneven, dirty, or slippery floors, along with objects on floors, to be more troublesome than the type of floor. This was especially true in factory settings and janitorial work, as there often was litter on the ground.

*Then there are certain areas where… the ground is unbelievably uneven then… then*,* you notice it [the pain] more.* (P9)

Physical work demands and time spent on the feet during work were perceived by the participants as affecting their foot pain. This was not always continuous time spent on the feet, and included switching between standing and sitting, as well as between standing and walking. Frequent use of stairs, long hours standing still, walking long distances, and heavy lifting were described as impacting foot pain. Many of the participants saw a direct relation between their workload, time spent on their feet, and foot pain.

The participants expressed a need to slow down the pace of work, and stressed the importance of taking sitting breaks whenever possible and getting help from others to finish work tasks. However, some participants felt that resting their feet periodically contributed to worsening their pain, especially when they resumed work, which led to a reluctance to take breaks and thus endure more pain than before the resting period.

*Well*,* I kind of try to sit down and rest. Instead of just standing*,* but like I said*,* it’s usually hard to get started again.* (P2)

Choice of footwear at work was thought to be “make or break” in relation to foot pain. Some of the participants worked in fields that required special, steel-reinforced shoes for workplace safety, and described these as hard, unergonomic, and uncomfortable. They understood their importance for safety, but wished for more individually oriented solutions. Others were able to wear more comfortable shoes, such as trainers or boots, at work. Regardless of mandated footwear, the importance of “good shoes” was recognised. Throughout the interviews, the participants expressed a desire to acquire what they believed to be good shoes to alleviate pain while working, if they had not already purchased then.

*Oh yes*,* in this case*,* what you can do is be careful choosing shoes. Yes. I mostly wear trainers now*,* shoes that are a bit more constructed and stable. [… ] No… no Crocs like others do. […] Or Birkenstocks… No*,* those? Those are really difficult shoes.* (P10)

#### Feeling like just another cog in the machine due to limited control over workplace organisation

Participants stated that altering the manner in which they performed required work tasks was easier than modifying the tasks themselves, despite these tasks being perceived as triggers of foot pain in the workplace. Changing work tasks to better suit needs and alleviate pain was described as difficult in relation to work requirements. It was easier to change work habits, such as the pace of work and the time of the day when certain tasks were completed. In each profession hindrances were perceived, as workload was often described as being dependent on a schedule or the nature of the tasks, for example not being able to complete a work task without strain on the feet. Recovery was described as only available during time off work, in evenings, and at weekends.

*Yes*,* I was limping for a while*,* but I still tried to walk normally*,* because otherwise it’s something else that starts to hurt. I still tried. Just slower. I maybe didn’t walk as fast. Tried to reduce speed. When there were long straight paths*,* I didn’t walk as fast.* (P8)

When they were asked whether they had mentioned their foot pain at work, the participants expressed that they had discussed this and its impact on work with their colleagues and/or employers. More often than not, this conversation had led to colleagues admitting to work-related foot pain themselves, as well as offers to help when needed. Where the participants worked as a part of a team, co-workers tried often to create opportunities for rest for the participants.

Those who did not work as part of a team usually had not mentioned their foot problems at work. This was ascribed to feeling that there were more important issues to discuss, or to a fear of appearing to complain too much. When these participants were asked if they had mentioned their foot pain to their employers, the answers tended towards the negative. Their reasoning varied from not wanting to be a burden to not wanting to seem ungrateful, and feeling that it would be meaningless and not lead to change.

*I: Have you talked to your employer about your foot problems?*

*P: No.*

*I: Why not?*

*P: There isn’t any point to take it up [with my employer]. It’s my own responsibility.* (P3)

#### Experiencing an uphill battle due to inequality and low quality of life outside of work

This sub-theme describes factors that were not dependent on work, but were still perceived as connected to experiences of pain in the feet. This includes experiences of impact on family life and the mental toll of living and working with foot pain, as well as expectations of medical treatment.

The participants described foot pain impacting their leisure activities, explaining that foot pain had significant consequences for their participation in social activities and hobbies. It often prevented them from participating in activities that might trigger foot pain, such as walks, playing with their children, spending time with their family and friends, and undertaking physical activity. The participants described often choosing to either distance themselves from these settings, or participate for less time than they otherwise would. Pain also impacted how household tasks were taken care of, as rest was often needed after work in order for them to be able to then manage everyday household tasks such as cleaning and cooking for the family.

*When the pain was at its worst and people wanted to go for walks and such*,* that’s when I was reluctant to go. Because I knew I had to pay for it afterwards*,* I felt… I needed to rest and save my feet.* (P10)

Throughout the interviews, the participants expressed a sense of resignation regarding their situations. Statements describing ideas such as needing to “grit my teeth and keep going” were common. There was a general sense of hopelessness and fear of never recovering and recurring pain – that the work that caused the pain often would not change, but also as justification of pain being a sign of achievement. A fear of movement in general was expressed, as the participants did not want to aggravate the pain, as well as a belief that pain was a part of working in their field.

*I often don’t think so much about the pain itself when I am at work*,* it’s more important to… to do the work*,* and then*,* like*,* when one starts to be done*,* then I feel that “damn*,* now my feet hurt. Now I have worked”.* (P4)

The participants also discussed the psychological impact that constant pain had on them. In many cases, pain impacted sleep patterns negatively, with the participants describing waking up repeatedly throughout the night. The participants expressed that pain negatively affected their mood and general feeling of wellbeing, which resulted in negative thought patterns towards the workplace, work tasks, and colleagues. They were also easily irritated, sad, and lacked energy, even outside of work:

*And when one doesn’t have the energy or capability one gets angry and sad because of the damned pain.* (P7)

The participants reported impacts on their personal lives, most importantly in terms of their partners and families. Partners had to shoulder more responsibility at home, which resulted in feelings of guilt and inadequacy on the part of the participants. Not being able to play or be physically active with their children affected their mood and sense of self-worth negatively, and made them feel unable to fulfil their roles as partners and parents.

The participants who had sought medical help had often done so when the pain had been unchanged for long periods of time, or when they felt that they could no longer perform adequately at work. The participants who had not sought medical attention, even though they experienced pain that impacted their work life, often did not trust healthcare in general, doubting that help would be given for their specific problem, or lacked the motivation or energy to contact a healthcare provider.

*I know that I probably would benefit from going to the doctor*,* but you need to have the energy to do that*,* too.* (P4)

The treatment provided by physiotherapists ranged from specific foot exercises to passive treatment such as extracorporeal shockwave therapy or shoe inserts. The participants often had more confidence in passive treatments, insoles, and better shoes than exercises. Regardless of whether they had tried exercises, the participants expressed doubts whether exercises would benefit them at all; they often experienced increased discomfort while performing exercises, which confirmed the belief that activity in any form would worsen foot pain, thus leading to inadequate compliance. However, some participants expressed a strong belief in the benefit of exercises as treatment, and that the only reason they were able to manage their pain was regular physical activity and good compliance with prescribed exercises.

## Discussion

The main findings suggest that foot pain leads to negative experiences and struggles, during both work and leisure time. Participants perceived their pain as being influenced by physical work demands and workplace conditions, while limited control over tasks and organisation contributed to negative experiences. Foot pain also restricted leisure and household activities and affected emotional well-being. Although some linked their pain to previous injuries, most believed that work caused or worsened their symptoms and that support was mainly available outside the workplace.

In the sub-theme `Struggling with increased pain due to work conditions and environment´ two work-environment factors were consistently described as having a negative impact on foot pain – certain types of floors, and long hours working and standing. In line with that, Werner et al. [22] reported that prolonged standing and walking increase the risk of plantar fasciitis by 52%, with an additional 30% risk from standing on hard surfaces [22]. Another study on workplace musculoskeletal pain highlights prolonged standing as an important factor [8], suggesting that hard floors can increase the risk of developing pain in the feet. Furthermore, our participants felt that choice of footwear was an important part of managing, or even minimising, the perceived negative effect of certain floor types on their feet. Access to the “right” footwear was considered to be essential for foot health. This is supported by research indicating that unsuitable footwear is more detrimental to lower-limb pain than hard floors. While soft mats show minimal benefit, softer shoes effectively reduce swelling, for example [32]. A narrative review that investigated the relation between work factors and lower-extremity musculoskeletal disorders found mixed results regarding floor type, while footwear – particularly softer shoes – was associated with reduced fatigue, pain, and swelling [33]. Footwear, types of floors, and work arrangements that require prolonged standing and walking may all contribute to foot pain and discomfort, emphasizing the importance of employer support and workplace interventions.

The sub-theme also highlights how various work tasks, in addition to prolonged standing and walking, may contribute to foot pain. Tasks such as heavy lifting, frequent climbing of stairs, jumping, and carrying objects for long periods of time were experienced as increasing foot pain. The participants described adapting some tasks using lifting aids, and avoiding stairs using the lift. Consistent with these findings, a systematic review found that weight-bearing tasks and standing or walking on hard floors increased the risk of developing plantar fasciitis [34]. As several of the included studies were of low methodological quality, further research is needed to strengthen the evidence regarding the relationship between foot pain and different work tasks across occupational settings.

In the sub-theme ‘Feeling like just another cog in the machine due to limited control over workplace organisation´, the participants described limited ability to influence work, and how tasks were performed also had an impact, as it was believed to be generally easier to change work habits than to change the work itself. Other studies confirm that pain in the lower limbs can negatively impact ability to participate in work and complete work tasks. Where occupations involve prolonged standing or walking and heavy lifting, study participants describes being unable to perform their work tasks [12–16]. This can lead to sick leave, and a lower income as a consequence [15]. This was also reflected in our study, where participants described a resolute mindset and feeling compelled to continue working despite experiencing pain. While concerns about job security may discourage workers from requesting workplace adjustments, supportive work environments and a sense of inclusion appeared to facilitate continued work participation despite ongoing symptoms. Additional research is needed to further understand how various work-related factors and environmental conditions impact the experience and development of work-related foot pain. Such research is essential to establish a foundation for improving the workplace condition and developing practical, evidence-based interventions and preventative measures.

The sub-theme ‘Experiencing an uphill battle due to inequality and low quality of life outside of work´ highlights how work-related foot pain negatively affected participants’ personal lives, limiting household, family, and leisure activities. Other research shows that individuals who suffer from foot or ankle pain often experience limitations in participating in activities such as walking, running, prolonged standing, walking down stairs, and physical activity in general [12–16], which mirrors the experiences expressed in our study. Studies show that loss of mobility can affect the independence of individuals, who can require help in daily-life activities such as housework [14–16], as expressed by the participants in this study. There is also evidence that recreational activities such as going on holiday and interacting with one’s children can be impacted [12, 14]. Furthermore, pain and difficulty participating in tasks and activities at home can negatively impact an individual’s mental health. Studies show that foot pain made it difficult to participate in social activities which often led to a feeling of social isolation [12, 15]; some even expressed a loss of the sense of self [16]. Research shows that pain in the foot, and loss of activity linked to this, are often linked to negative emotions, low mood, fear of physical activity, depression, anxiety, frustration, and trouble sleeping [12–16]. In addition to difficulties performing work tasks, participants reported reduced participation in leisure activities, exercise, household chores, and family life. This study indicates a significant impact of foot pain on private life and mental health, relating to feelings of guilt, pressure to participate in family or leisure activities, negative thought patterns. These factors and other psychological factors need to be considered in relation to work-related foot pain. Employers and healthcare providers can use this knowledge to offer more person-centred treatments, and better support individuals in managing the impact of pain at work and on their overall well-being. As the topic is not largely researched but shows potential to increase the quality of life of workers, further studies need to be conducted.

Participants’ beliefs about and experiences of treatment varied. While physiotherapist-prescribed exercises were generally perceived as effective, some participants preferred rest or passive treatments. Similar findings have been reported in patients with knee osteoarthritis, where misconceptions about exercise led to preferences for passive treatment or surgery [35]. A lack of confidence in provided treatments and exercises has been reported in multiple studies [15, 16], as have a desire for trustworthy educational resources that are available to the public and clear, consistent information from healthcare providers. Such information helps patients to understand the pathologies behind their pain, and to manage expectations regarding recovery [12, 13, 15, 16], and a desire for this was also expressed in our study.

The participants described shoe insoles as one treatment option for alleviating symptoms, and often found their usage to be a suitable strategy for minimising pain and continuing to work. Individuals with foot pain often use trial-and-error methods, such as different footwear, shoe insoles bought over the counter, and taping of the feet. In some cases, individuals try behavioural modification before they seek professional help [12]. The effects of shoe insoles have been confirmed by Ramos-Petersen et al., who showed that orthopaedic orthoses improved wellbeing and activity levels in patients with rheumatoid arthritis over six months [36].

The interviews highlighted the importance of understanding foot pain and its causes. The participants lacked knowledge on how to avoid exacerbating their pain, the treatment options available to them, and how recommended treatments could help their recovery. Cotchett et al. highlighted the need for improved education on foot pain and treatment options, as conflicting information from online sources and healthcare professionals often led to frustration and confusion [12]. Patients perceived not being listened to by healthcare professionals [12], and some individuals preferred a more hands-on approach [12, 16]. Patients with chronic pain often describe having tried several forms of treatment, each with varying effects [37]. More accessible information on treatment and education regarding pain, as well ensuring that individuals feel that they are being taken seriously, may help to improve treatment compliance and prevent further pain.

### Methodological considerations

The trustworthiness of our results needs to be considered. The method of semi-structured interviews based on an interview guide was well suited to the aim of the study, and gave rich data to analyse. In the interviews participants were urged to speak freely with their own words. Eventually answers were probed with follow up questions to expand and provide clarifications. It created room to explore individuals’ experiences [28]. A limitation of this study may be the relatively small number of participants and the purposive sampling. The participants in this study were predominantly women, with only three men. Therefore, the results of the study may not adequately represent men’s experiences. The small number of men can be explained by women being more likely to report pain, and therefore more likely to seek medical attention for their pain, than men [5, 7]. Another limitation of this study is the fact that the interviews were conducted by a total of four data collectors, with different levels of interviewing and clinical experience, although it should be noted that an interview guide was used to mitigate this. Participants were given the choice of being interviewed either digitally or face-to-face. One interview was conducted digitally, via Zoom; the others were conducted in one-to-one meetings. This may have affected the richness of the data collected between the interviewers and interview settings, and may have led to interviewer bias [38, 39]. By choosing not to recruit any of the authors’ own patients, a conscious effort was made to minimise the risk of interviewer bias [40].

QCA is a widely used and well-established qualitative analytical method [23]. Our interview guide, the coding of interviews and the final results were discussed between all authors. Triangulation between authors was used, which may have contributed to a more nuanced discussion and analysis since the authors have a diverse background and experience.

The fact that the participants were employed in a wide range of professions may have positive implications regarding transferability in regards to people of working age, especially in professions who rely on long hours walking and standing, although the study may have missed an opportunity to recruit participants from a broader range of fields or backgrounds than the ones presented [41]. Further research with a less homogenous sample may be required to achieve a broader understanding of how foot pain impacts both genders.

## Conclusion

This study highlights that work-related foot pain is an important health issue that profoundly impacts the lives of individuals, both professionally and personally, and often results in a sense of resignation. The experiences described by the participants shine a light on how individuals suffering from pain try to work around it, as well as how work-related pain affects social and family life and creates negative emotions. An understanding of how foot pain can impact individuals in their work lives may help to reduce these factors. The results of this study show the importance of identifying work-related factors, and could be used by employers when considering preventative measures, to minimise injuries or sick leave related to discomfort in the feet.

## Supplementary Information

Below is the link to the electronic supplementary material.

Supplementary Material 1

## Data Availability

The datasets generated and analysed during the current study are not publicly available due compromise participants anonymity. Selected parts of the interviews, coding examples, additional quotations, and other relevant data are available from the corresponding author on reasonable request.

## Abbreviations

I: interviewer
ICF: International Classification of Function, Disability and Health
P: participant
QCA: Qualitative Content Analysis
SEFAS: Self-reported Foot and Ankle Score
VAS: Visual Analogue Scale
WMSDs: Work-related musculoskeletal disorders
