# Supplementary material for: The impact of work-related foot pain on work and personal life: an interview study
Source: BMC Musculoskelet Disord. 2026 Oct 14;27:918. doi: 10.1186/s12891-026-10624-3 (PMC13711864; doi:10.1186/s12891-026-10624-3)
Supplement: Supplementary file 1 [file 12891_2026_10624_MOESM1_ESM.docx]

## Supplementary - Interview Guide

**Opening question:**
Can you describe your foot pain and how it relates to your work?

**Body Structure and Function**

- Can you describe your experience of having problems with your foot or feet?
- How did your symptoms started, and in what context did the symptoms first occur?
- Have your foot problems changed over time?

**Activity**

- How do your symptoms vary throughout the day? Do you experience any daily variations?
- How are your symptoms affected by your work and daily work activities?
- Are there specific tasks or activities at work that you believe influence your foot problems?
- Which work-related tasks have the greatest impact on your foot problems?
- Are there any work tasks or situations that aggravate your foot symptoms particularly?

**Participation**

- In which situations do you experience the greatest strain on your foot?
- Have you modified any work tasks or activities because of your foot problems? If so, how?
- Have you changed your workday in any way because of your symptoms?
- Are there any changes you would like to make to your workday or work tasks to better manage your foot symptoms?

**Environmental Factors**

- How does your workplace facilities or work environment affect your foot symptoms?
- How do workplace equipment or other aspects of the workplace influence your symptoms?
- What opportunities do you have for recovery, autonomy in your work, and variation in your work

**Personal Factors**

- Do you primarily work independently or as part of a team?
- **Follow-up question:** Do your foot problems affect your interactions or collaboration with colleagues?

**Additional Questions**

- Are there other people in your workplace who also experience foot problems?
- Have you discussed your foot symptoms with your employer?
  - **If no:** Could you tell me why not?
  - **If yes:** Has your employer taken your symptoms into account? If so, in what way?
- Has your employer planned or implemented any measures or workplace accommodations to help manage your symptoms?
- Do your leisure-time activities or hobbies have any influence on your foot symptoms?
- How would you describe your level of physical activity?
- Have you sought healthcare or treatment for your foot symptoms?
- Are you currently performing any exercises or training for your feet?
  - **Follow-up question:** Can you describe what this involves?

**Closing question:**
Before we finish, is there anything else about your foot symptoms, your work, or your experiences that you think is important for us to understand?
